# Auriculotherapy for sleep quality in people with primary insomnia: A protocol for a systematic review and meta-analysis

**DOI:** 10.1097/MD.0000000000014621

**Published:** 2019-02-22

**Authors:** Runyuan Ren, Jiayuan Zhang, Tingting Zhang, Yangzhi Peng, Chenjian Tang, Qi Zhang

**Affiliations:** Chengdu University of Traditional Chinese Medicine, Chengdu, Sichuan Province, China.

**Keywords:** auriculotherapy, meta-analysis, primary insomnia, randomized controlled trial, systematic review

## Abstract

**Background:**

Primary insomnia is one of the common sleep disorders. Auriculotherapy originated from traditional Chinese medicine, has been thought as a promising treatment for primary insomnia. We aim to evaluate the efficacy and safety of auriculotherapy for patients with primary insomnia through this systematic review.

**Methods:**

Five English databases (Cochrane Central Register of Controlled Trials, MEDLINE, EMBASE, AMED, and CINAHL), 4 Chinese databases (CBM, CNKI, CQVIP, and Wanfang), and 5 clinical trial registration databases (ClinicalTrials.gov, ANZCTR, EU-CTR, ChiCTR, and ICTRP) will be searched from establishment of the database until November 2018. Articles written in English or Chinese languages will be included. The randomized controlled trials of auriculotherapy (auricular acupuncture and auricular acupressure) for patients with primary insomnia will be included. The primary outcome will be assessed according to the Pittsburgh sleep quality index. Meta-analysis will be conducted with the use of RevMan 5.3. The specific process will refer to the Cochrane Handbook 5.1 for Systematic Review.

**Results:**

High-quality synthesis of current evidence on the efficacy and safety of auriculotherapy for primary insomnia will be provided in this study.

**Conclusion:**

This systematic review aims to present evidence for whether auriculotherapy is an effective intervention which can improve sleep quality in patients suffering primary insomnia.

**PROSPERO registration number:**

CRD42019106422.

## 1 Introduction

Insomnia is one of the most prevalent sleep disorders, which is experienced by approximately 30% to 50% of the general population.^[1]^ It has been shown that sleep difficulties are more common in women and older adults,^[2–4]^ which also be a relevant symptom in childhood and adolescence with a prevalence of approximately 20%.^[5]^ In France, 1 in 5 persons suffers from primary chronic insomnia.^[6]^ In the USA, the number of office visits with insomnia as the stated reason for visit increased 13% from 1999 to 2010.^[7]^

Insomnia, pain, and psychological distress are often intertwined.^[8]^ Current researches have indicated that the second-hand smoke exposure^[9]^ and stress^[10]^ may be the potential risk factors of sleep disturbance. And less positive emotions and greater negative content characterize the dreams of patients with primary insomnia.^[11]^

Since sleep is essential to the organism homeostasis,^[12]^ poor sleep quality predicts the incidence of several negative physical and mental conditions such as depression, anxiety, fibromyalgia, rheumatoid arthritis, whiplash, arthrosis, osteoporosis, headache, asthma, and myocardial infarction.^[13]^ Insomnia is also associated significantly with the presence of angina, hypertension, diabetes, obesity, stroke, fatigue, and subjective memory impairment.^[2,14,15]^ Besides, primary insomnia remains a significant predisposing factor for developing dementia.^[16]^

Recent studies on the etiology and pathogenesis of primary insomnia mainly involve the disruptions of functional brain networks. Abnormal intrinsic functional hubs in the left inferior frontal gyrus, middle temporal gyrus, and the right precuneus have been detected.^[17]^ Also, primary insomnia has been associated with the abnormal anatomical network architecture,^[18]^ disturbed striatal functional connectivity with the default mode network, the visual and somatosensory areas^[19]^ and a decrease in resting-state functional connectivity and structural connectivity of right superior temporal pole, left middle temporal, and left inferior occipital regions.^[12]^

Treatment options for insomnia mainly include cognitive behavioral therapy (CBT), behavioral therapy or pharmacotherapy.^[20]^ Benzodiazepines and benzodiazepine receptor agonists have been widely applied in clinical practice. However, because of the side effects, long-term efficiency and tolerability of pharmacotherapy,^[5,21]^ non-drug treatment strategies such as CBT are preferred. CBT is effective for enhancing the overall quality of sleep and reducing the symptoms of insomnia disorder but is hindered by cost and limited access to treatment.^[22,23]^ Hence, a growing number of insomnia sufferers are seeking for complementary and alternative therapy which is cheaper, more convenient and has less adverse effects.^[24,25]^

Auriculotherapy, originated from ancient Chinese medicine, which includes the modalities of auricular acupuncture and ear acupressure by stimulation of specific acupoints on the external ear. Since it is thought that different auricular regions correspond to particular somatotopic areas of the body,^[26]^ auriculotherapy has been used as an alternative non-pharmacological treatment for certain internal diseases including insomnia.^[27–30]^

As the clinical reports on auriculotherapy for primary insomnia have gradually increased in the last few years, a high-quality systematic review is still lacking. Therefore, we conduct this systematic review to objectively evaluate whether auriculotherapy is a more effective and safer therapy for primary insomnia.

## 2 Methods

### 2.1 Registration

This systematic review protocol has been registered on PROSPERO as CRD42019106422. In this paper, the protocol will be performed using the methods introduced in the Cochrane Handbook 5.1 for Systematic Reviews of Intervention 16 and reported according to the PRISMA-P guidelines17. If we will refine procedures described in this protocol, we will document the amendments in the PROSPERO database and disclose them in future publications related to this meta-analysis.

### 2.2 Eligibility criteria for considering studies

#### 2.2.1 Types of studies

The randomized controlled trials (RCTs) were eligible. Articles not written in English or Chinese languages and repeatedly published articles should be excluded.

#### 2.2.2 Types of participants

Participants of any age, gender, or ethnic background diagnosed with primary insomnia are included. The standard diagnostic instruments include diagnostic and statistical manual of mental disorders,^[31]^ Chinese classification and diagnosis of mental disease,^[32]^ and other recognized diagnostic criteria.

Participants with comorbid disorders are excluded. Participants with other sleep disorders, such as parasomnias, narcolepsy, delayed sleep phase type of circadian rhythm sleep–wake disorders, breathing-related sleep disorders, and restless leg syndrome are excluded. Baseline is uniform for all participants in each RCT.

#### 2.2.3 Types of interventions

The experimental group should be treated with auriculotherapy including auricular acupuncture or auricular acupressure, and acupoints used according to TCM nomenclature. Auriculotherapy combined with medication or other treatment should be excluded.

The control group should adopt one of the following treatment methods: Sham auricular acupuncture or acupressure, placebo, medication (benzodiazepines or non-benzodiazepines), or no treatment. The treatment duration is unlimited. Nursing measures should be consistent between the 2 groups.

#### 2.2.4 Types of outcome measures

##### 2.2.4.1 Primary outcome

Overall sleep quality will be measured by the Pittsburgh sleep quality index (PSQI). The higher the global PSQI score is, the worse the sleep quality is.

##### 2.2.4.2 Secondary outcome

(1) Frequency and nature of adverse events,
(2) The total scores of the insomnia severity index,
(3) The total scores of the Athens insomnia scale, and
(4) Objective sleep parameters such as sleep-onset latency, total sleep duration, sleep efficiency (ratio of time asleep to time on bed), and frequency of early awakenings measured by PSG, actigraphy, CardioPulmonary Coupling, ambulatory electroencephalogram, or self-assessment sleep quality tools (sleep diary).

### 2.3 Search methods for identifying the studies

#### 2.3.1 Data sources and searches

Five English databases (Cochrane Library, MEDLINE, EMBASE,CINAHL, and AMED), 4 Chinese databases (CBM, CNKI, CQVIP, and Wanfang), and 5 clinical trial registration databases (ClinicalTrials.gov, ICTRP, ChiCTR, EU-CTR, and ANZCTR) will be searched from establishment of the database until November 2018. Only articles written in English or Chinese languages are eligible.

The key search terms are ([“sleep initiation and maintenance disorders” OR “DIMS” OR “early awakening” OR “insomnia∗” OR “sleep initiation dysfunction” OR “Sleeplessness”] AND [“auricular acupuncture” OR “auricular acupressure” OR “auricular pressing” OR “auricular needle” OR “auricular plaster” OR “auricul∗” OR “auricular acupoint∗” OR “auricular point∗” OR “otopoint∗” OR “ear point∗”]).

### 2.4 Study selection and data extraction

**Figure 1:**
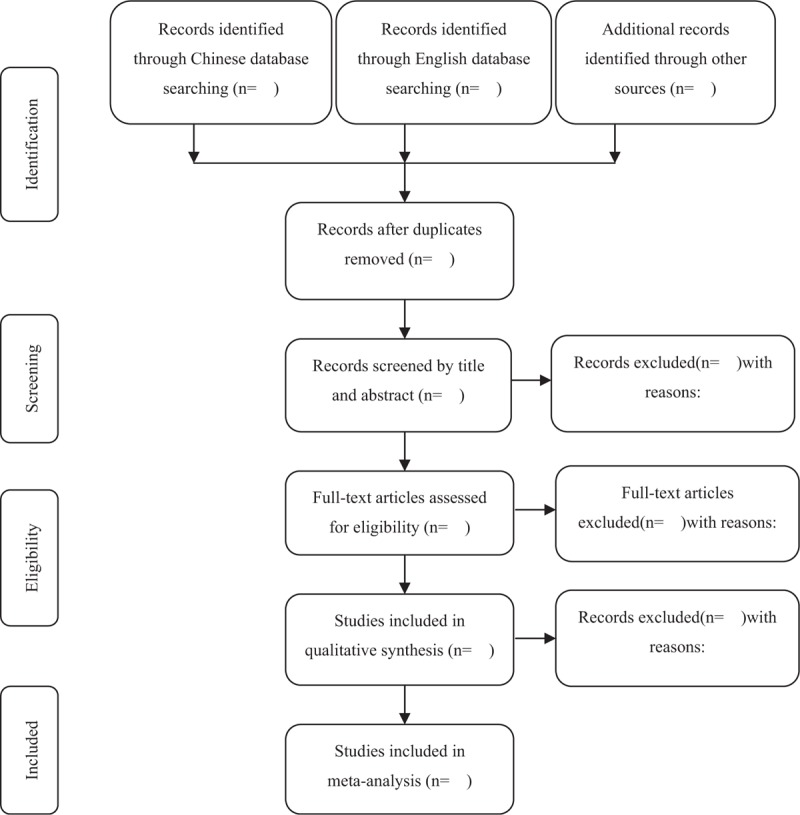
Flow diagram of the study selection process.

Two researchers (RuR and YP) search and screen the studies independently by finding duplications, excluding irrelevant titles and abstracts, and then selecting eligible studies by reviewing full texts. The inclusion and exclusion criteria are listed above. Reasons for exclusion should be noted. The third reviewer (TZ) verified all information. Any disagreements should be solved by discussion until a consensus was reached. The specific process of study selection is shown in Figure 1.

Data extraction will be performed by 2 review authors (RR and YP) independently. The data extraction is conducted by a standard form, which contained first author, characteristics of the study type (date of publication, country, and arm), participants (sample size, mean age, gender, and duration of insomnia history), diagnostic instrument, intervention details, outcomes, follow-up, adverse events, and reported results. Finally, the data obtained by the 2 reviewers will be checked each other. If the data is incomplete, the original author will be contacted.

### 2.5 Assessment of risk of bias

Two researchers (RR and YP) assess the risk of bias independently, using a collaboration tool recommended by the Cochrane Handbook 5.1.^[33]^ Seven domains should be evaluated, including random sequence generation, allocation concealment, blinding of participants and personnel, blinding of outcome assessment, incomplete outcome data, selective reporting, and other bias. Disagreement will be settled by discussion.

### 2.6 Data analysis

#### 2.6.1 Date synthesis

Data analysis will be performed with Review Manager 5.3 software provided by the Cochrane Collaboration (www.cochrane.org). Continuous outcomes were presented as mean difference with 95% confidence interval (CI) between 2 groups, whereas dichotomous data were presented as relative risk with 95% CI. It is considered statistically significant when *P* < .01.

#### 2.6.2 Assessment of heterogeneity

The chi-square test and *I*^2^ statistic are used to assess heterogeneity. The fixed-effect model is suitable to estimate the typical effect for studies with low heterogeneity (*I*^2^ < 50% or *P* > .10), whereas the random-effects model is used to assess the average distribution for studies with substantial unexplained heterogeneity (*I*^2^ ≥ 50% or *P* ≤ .10).

#### 2.6.3 Subgroup analysis and sensitivity analysis

Subgroup analysis and sensitivity analysis will also be employed to explore the possible causes of heterogeneity. Subgroup analysis will be based on possible factors that may lead to heterogeneity, such as intervention (auricular acupuncture and auricular acupressure), control (medication and sham auriculotherapy or placebo), ages (young, middle, and old), treatment duration, the quality of study, and so on. Narrative synthesis will be considered if quantitative synthesis is not appropriate.

### 2.7 Assessment of publication bias

If more than 10 articles are included, publication bias will be analyzed by visual inspection of funnel plots. A symmetrical distribution of funnel plot data indicates that there is no publication bias.

### 2.8 Confidence in cumulative evidence

GRADE system will be used for assessing the strength of the body of evidence.^[34]^ According to the grading system, the quality of evidence will be rated high, moderate, low, and very low.

## 3 Discussion

Primary insomnia is the second most common mental disorder, which is highly comorbid with other psychological and medical disorders.^[1]^ Complementary and alternative therapies have been accepted by an increasing number of persons suffered from sleep disorders for certain advantages like convenient, cheap, and less side effects.

As one of the complementary and alternative therapies, auriculotherapy has been widely applied in clinic especially in Asian countries. Since several recent clinical researches have focused on this promising treatment for primary insomnia, it is necessary to perform a high-quality systematic review and meta-analysis. Therefore, this review is expected to provide rigorous and objective evidence of the efficacy and safety of auriculotherapy for primary insomnia.

## Author contributions

Runyuan Ren and Qi Zhang contributed to the conception of the study. The manuscript of the protocol was drafted by Runyuan Ren and Chenjian Tang, which was revised by Qi Zhang and Jiayuan Zhang. The search strategy was developed by all authors and run by Runyuan Ren and Yangzhi Peng, who will also independently screen the potential studies, extract data of included studies, assess the risk of bias and finish data synthesis. Tingting Zhang will arbitrate the disagreements and ensure that no errors occur during the study. All authors have approved the publication of the protocol.

**Conceptualization:** Runyuan Ren, Jiayuan Zhang, Chenjian Tang, Qi Zhang.

**Conceptualization:** Runyuan Ren, Qi Zhang, Jiayuan Zhang, Chenjian Tang.

**Data curation:** Runyuan Ren, Tingting Zhang, Yangzhi Peng.

**Data curation:** Runyuan Ren, Yangzhi Peng, Tingting Zhang.

**Formal analysis:** Jiayuan Zhang, Runyuan Ren.

**Formal analysis:** Runyuan Ren, Jiayuan Zhang, Yangzhi Peng.

**Investigation:** Runyuan Ren.

**Methodology:** Runyuan Ren, Jiayuan Zhang.

**Project administration:** Tingting Zhang, Qi Zhang.

**Resources:** Yangzhi Peng.

**Software:** Runyuan Ren, Tingting Zhang, Yangzhi Peng.

**Supervision:** Tingting Zhang, Qi Zhang.

**Validation:** Qi Zhang.

**Writing – original draft:** Runyuan Ren, Jiayuan Zhang, Chenjian Tang, Qi Zhang.

**Writing – review and editing:** Runyuan Ren, Jiayuan Zhang, Qi Zhang.
